# Fasting insulin sensitivity indices are not better than routine clinical variables at predicting insulin sensitivity among Black Africans: a clamp study in sub-Saharan Africans

**DOI:** 10.1186/1472-6823-14-65

**Published:** 2014-08-09

**Authors:** Eugene Sobngwi, Andre-Pascal Kengne, Justin B Echouffo-Tcheugui, Simeon Choukem, Joelle Sobngwi-Tambekou, Eric V Balti, Mark S Pearce, Valentin Siaha, Aissa S Mamdjokam, Valery Effoe, Eric Lontchi-Yimagou, Oliver T Donfack, Barbara Atogho-Tiedeu, Philippe Boudou, Jean-Francois Gautier, Jean-Claude Mbanya

**Affiliations:** 1 Department of Internal Medicine, Faculty of Medicine and Biomedical Sciences, University of Yaounde I, Yaounde, Cameroon; 2 National Obesity Centre, Yaounde Central Hospital, Yaounde, Cameroon; 3 South African Medical Research Council & University of Cape Town, Cape Town, South Africa; 4 The George Institute for Global Health, Sydney, Australia; 5 Hubert Department of Global Health, Rollins School of Public Health, Emory University, Atlanta, Georgia, USA; 6 Faculty of Health Sciences, University of Buea, Buea, Cameroon; 7 Department of Internal Medicine, Douala General Hospital, Douala, Cameroon; 8 Centre of Higher Education in Health Sciences, Catholic University of Central Africa, Yaounde, Cameroon; 9 Diabetes Research Center, Brussels Free University-(VUB), Brussels, Belgium; 10 Institute of Health & Society, Newcastle University, Newcastle upon Tyne, UK; 11 Wake Forest Institute for Regenerative Medicine, Wake Forest University, Winston-Salem, North Carolina, USA; 12 Laboratory of Molecular Medicine and Metabolism, Biotechnology Centre, Nkolbisson, University of Yaounde 1, Yaounde, Cameroon; 13 Unit of Transfer in Molecular Oncology and Hormonology, Saint-Louis University Hospital, Assistance Publique - Hôpitaux de Paris, Paris, France; 14 Department of Diabetes and Endocrinology, Saint-Louis University Hospital, Assistance Publique - Hôpitaux de Paris, University Paris-Diderot Paris-7, Paris, France

## Abstract

**Background:**

We aimed to evaluate the predictive utility of common fasting insulin sensitivity indices, and non-laboratory surrogates [BMI, waist circumference (WC) and waist-to-height ratio (WHtR)] in sub-Saharan Africans without diabetes.

**Methods:**

We measured fasting glucose and insulin, and glucose uptake during 80/mU/m^2^/min euglycemic clamp in 87 Cameroonians (51 men) aged (SD) 34.6 (11.4) years. We derived insulin sensitivity indices including HOMA-IR, quantitative insulin sensitivity check index (QUICKI), fasting insulin resistance index (FIRI) and glucose-to-insulin ratio (GIR). Indices and clinical predictors were compared to clamp using correlation tests, robust linear regressions and agreement of classification by sex-specific thirds.

**Results:**

The mean insulin sensitivity was M = 10.5 ± 3.2 mg/kg/min. Classification across thirds of insulin sensitivity by clamp matched with non-laboratory surrogates in 30-48% of participants, and with fasting indices in 27-51%, with kappa statistics ranging from −0.10 to 0.26. Fasting indices correlated significantly with clamp (/r/=0.23-0.30), with GIR performing less well than fasting insulin and HOMA-IR (both p < 0.02). BMI, WC and WHtR were equal or superior to fasting indices (/r/=0.38-0.43). Combinations of fasting indices and clinical predictors explained 25-27% of variation in clamp values.

**Conclusion:**

Fasting insulin sensitivity indices are modest predictors of insulin sensitivity measured by euglycemic clamp, and do not perform better than clinical surrogates in this population.

## Background

Type 2 diabetes mellitus (T2DM) and obesity, which are both associated with insulin resistance, are increasingly common worldwide, especially in developing countries including sub-Saharan African (SSA) [1,2]. Furthermore, the highest relative increases in diabetes (by 90%) prevalence by 2030 is projected to occur in SSA [3]. The experienced increasing trends of T2DM and obesity over the last two decades in SSA have revealed new challenges for diagnosis, prevention and treatment, as well as uncovering context specific causes and promoters of the conditions. Given the central role of insulin resistance in the pathogenesis of T2DM and obesity, measures of insulin sensitivity are very important diagnostic and research tools. The gold standard for assessing insulin sensitivity is the hyperinsulinemic-euglycemic clamp, which measures the in vivo rate of insulin-stimulated glucose uptake [4]. However, this method is invasive, costly, logistically challenging, technically demanding, and time consuming. Consequently, it is impractical and not applicable in large-scale epidemiological and intervention studies, especially in resource-poor settings of SSA. Surrogate indices of insulin sensitivity that can be used in large-scale studies have been developed [5], and validated in several populations [5], mainly Caucasians. However, there are suggestions that ethnicity may influence the performance of available surrogate indices of insulin sensitivity, particularly among populations with high prevalence of insulin resistance such as Africans [6,7]. This issue however, remains largely under-investigated and little is known on the diagnostic utility of common insulin sensitivity indices, and whether they even do better than non-laboratory-based determinants of insulin sensitivity, in SSA populations. We therefore assessed the performance of common fasting indices of insulin sensitivity and clinical surrogates of insulin resistance, against hyperinsulinemic-euglycemic clamp-measured insulin sensitivity, among non-diabetic Cameroonians adults with a wide range of insulin sensitivity/resistance profile.

## Methods

### Study population

We recruited 87 non-diabetic volunteers of sub- Saharan African origin (51 men and 36 women) aged 21 to 61 years, from 2006 to 2008 in Cameroon. Eligible healthy subjects were recruited through the outpatient clinic of the National Obesity Center of the Yaounde Central Hospital [8]. Potential participants were screened by history, physical examination and biochemical tests. A fasting plasma glucose of ≥ 126 mg/dl (7 mmol/L) indicated diabetes [9]. Participants with serum creatinine concentrations >1.5 mg/dl, on medications that may impact on energy metabolism, with human immunodeficiency virus (HIV)-positive status, or with overt chronic liver, renal, or thyroid disease, active coronary artery disease, and smoking more than 20 cigarettes per day, were excluded.

All volunteers were examined on two consecutive days, each after 12-h overnight fast and controlled diet and activity for 7 days. Participants had a diet consisting of 50% carbohydrates, 30% lipids, and 20% proteins, and vigorous physical exercise was prohibited. On day 1, we performed anthropometric measurements and an oral glucose tolerance test (OGTT), and on day 2, we performed a euglycemic hyperinsulinemic clamp.

### Anthropometric measurements

For all participants, we measured height to the nearest 0.5 cm, and weight in light clothes to the nearest 0.1 kg, and calculated the body mass index (BMI) as weight in kg/height^2^ in m^2^. Individuals were categorized as lean (BMI < 25 kg/m^2^), overweight (BMI: 25–29.9 kg/m^2^) or obese (BMI ≥ 30 kg/m^2^). We measured waist and hip circumference to the nearest 0.5 cm and calculated the waist-to-hip ratio as well as waist-to-height ratio (WHtR). Total fat mass, fat-free mass, and percent fat were measured by dual energy X-ray absorptiometry (DEXA) with an absorptiometer (Hologic QDR-1000/W, Wilmington, MA, USA) with Whole Body V5.73 software (n = 19), or by bioimpedance (TANITA BC 420 MA, TANITA Corporation 1-14-2 Maeno-cho, Tabashi-ku, Tokyo-Japan) (n = 68).

Blood pressure was the mean of two measurements performed at least three minutes apart, in the right arm with the subject sited after a 15-min rest with an Omron recorder (manufacturer references).

### Fasting measurements

#### Oral glucose tolerance test

After a 12 h overnight fast, each participant underwent a 75-gram OGTT over 120 minutes. We collected whole blood samples from an antecubital vein at 0, 30, and 120 min for the determination of blood glucose, and serum insulin and the evaluation of glucose tolerance according to World Health Organization Criteria (WHO 1998 criteria) [9], and early phase insulin secretory response.

#### Euglycemic hyperinsulinemic clamp

Whole-body insulin sensitivity was evaluated with a 120-minute euglycemic hyperinsulinemic clamp technique [4]. After a 12-h overnight fast, at 8 am, a priming dose of insulin infusion (Actrapid 100 IU/ml; Novo Nordisk, Gentofte, Denmark) was administrated during the initial 10 minutes to acutely raise plasma insulin to the desired level, where it was maintained by a continuous insulin infusion at a rate of 80 mU/m^2^ body surface area per minute. Blood glucose was clamped at 100 mg/dl (5.5 mmol/l) for 100 minutes by infusing 20% glucose at variable rates according to blood glucose measurements performed at 5-min intervals (mean coefficient of variation of blood glucose was <5%). The mean value for the period from 80 to 100 min was used to calculate the rates of whole body glucose uptake. In the fasting state and at 80, 90, and 100 min, arterialized blood samples for the measurement of plasma insulin were performed.

#### Biochemical assays

We performed all assays twice using the same batch of kits in each case. Glucose was measured by the glucose oxidase method, and we used immunoradiometric assays (Bi-insulin IRMA [Bio-Rad, Marnes la Coquette, France] to measure insulin. The intra-assay coefficient of variation was 1.8-3.8% and the inter-assay coefficient of variation was 2.6-8.0% for insulin. Serum total cholesterol, high-density lipoprotein (HDL)-cholesterol and triglycerides were measured by means of standard enzymatic techniques. Low-density lipoprotein (LDL)-cholesterol was calculated using Friedwald’s formula [10].

#### Calculations of clamp-derived and surrogate indices of insulin sensitivity

Insulin mediated glucose uptake (M value) was calculated from the glucose infusion rate during the final 20 min of the glucose clamp as the rate of exogenous glucose infusion divided by the steady-state clamp insulin concentration, after accounting for differences between individuals in glucose space (by dividing the average group steady-state glucose by the individual steady state glucose), and expressed in mg/min/kg of body fat-free mass [4].

Calculations of fasting-derived indices were made using the mean of two fasting glucose and insulin concentrations before the start of the euglycemic clamp. The fasting glucose to insulin ratio (Glucose/Insulin ratio), fasting insulin resistance index (FIRI = fasting glucose × fasting insulin/25) [11], the Homeostasis Model Assessment (HOMA) for insulin resistance (HOMA-IR) [12,13], and the quantitative insulin sensitivity check index (QUICKI) [14] were calculated according to established methods. Given that HOMA-IR and FIRI assess insulin resistance as opposed to sensitivity, negative correlations with euglycemic clamp measure of insulin sensitivity would be expected.

### Statistical analysis

Data are presented as mean (standard deviation, SD) for continuous variables and as count and percentage for categorical variables. We compared groups (BMI and gender) using the Kruskal-Wallis test for continuous variables, and assessed the heterogeneity across gender and BMI subgroups through interaction tests. Continuous associations between indices of insulin sensitivity was assessed graphically with the use of correlation matrix, applying the Box-Cox [15] power transformations to improve the shape of the associations; then the “*Covariance Estimation for Multivariate t Distribution*” [16] methods was used to derived the correlation coefficients, while minimising the potential effects of outliers. The Steiger t test was used to compare correlation coefficients among indices of insulin sensitivity. Regression coefficients to indicate the size of the association of each of the indices with euglycemic clamp was derived from robust multiple linear regressions model that included each of the indices of interest, waist circumference and sex as independent variables. Agreement was explored by examining the proportion of subjects correctly classified by surrogate indices of insulin sensitivity, within sex-specific thirds of clamp-derived measure of insulin sensitivity as the reference. This was supplemented by a formal statistical testing using the kappa test to compare the distribution of participants across increasing thirds of QUICKI and glucose/insulin ratio, or decreasing thirds of age, BMI, waist circumference, WHtR, fasting insulin HOMA-IR and FIRI, vs. increasing third of clamp derived insulin sensitivity. The 95% confidence interval around kappa estimates was from two-sided bootstrap methods, based on 1000 replications. Analyses were carried out using SPSS version 17.0 for Windows (SPSS Inc., Chicago, IL) and R statistical software version 2.13.0 [13-04-2011], (The R Foundation for Statistical Computing, Vienna, Austria). The significance level was set at 0.05.

## Results

### Characteristics of the study population

The mean age (SD) was 34.6 (11.4) years and the mean BMI was 27.6 (6.5) kg/m^2^. Table 1 shows the general characteristics of the 87 study participants, across sex-specific BMI categories. In both men and women, there were significant differences in percent fat, waist circumference, hip circumference, WHtR and total-cholesterol across BMI categories, with always significant linear trends. Systolic and diastolic blood pressures, two-hour glucose and fasting insulin levels were significantly different in men (waist-to-hip ratio in women) across BMI categories, again, with significant linear trends, except for systolic blood pressure. In both genders, no significant differences across categories of BMI were noted for age, triglycerides, and cholesterol (HDL and LDL) levels. There was evidence of heterogeneity by sex and across BMI categories for waist-to-hip ratio (p = 0.04 for interaction) fasting and 2-hour glucose (both p ≤ 0.05), and marginally for fasting insulin (p = 0.06), but not for other characteristics (Table 1).

**Table 1:** Characteristics of the subjects by sex and BMI categories

	**Men**					**Women**					**p gender and BMI interaction**
	**Lean**	**Overweight**	**Obese**	**p Kruskal Wallis**	**p linearity**	**Lean**	**Overweight**	**Obese**	**p Kruskal Wallis**	**p linearity**	
N	23	18	10			14	9	13			
Age (years)	33.1 ± 11.4	36.9 ± 9.3	38.3 ± 9.7	0.23	0.15	31.3 ± 12.9	35.3 ± 16.0	34.2 ± 10.7	0.70	0.56	0.91
BMI ( kg/m2)	22.1 ± 1.4	27.7 ± 1.7	35.3 ± 7.4	<0.001	<0.001	23.0 ± 1.4	27.7 ± 1.5	36.3 ± 5.9	<0.001	<0.001	0.86
Percent fat (%)	14.2 ± 3.6	23.2 ± 6.3	33.6 ± 9.6	<0.001	<0.001	26.6 ± 6.0	32.5 ± 8.0	40.8 ± 5.3	<0.001	<0.001	0.28
Waist circumference (cm)	76.0 ± 4.5	92.8 ± 8.4	109.0 ± 12.8	<0.001	<0.001	78.0 ± 6.7	85.4 ± 7.7	102.6 ± 11.4	<0.001	<0.001	0.09
Hip circumference (cm)	87.1 ± 5.9	97.2 ± 6.1	117.1 ± 15.1	<0.001	<0.001	92.0 ± 9.1	106.9 ± 9.7	119.8 ± 13.3	<0.001	<0.001	0.59
Waist-to-hip ratio	0.89 ± 0.03	0.93 ± 0.04	0.92 ± 0.05	0.12	0.10	0.85 ± 0.06	0.80 ± 0.04	0.88 ± 0.08	0.04	0.40	0.04
Waist-to-height ratio	0.44 ± 0.03	0.53 ± 0.05	0.63 ± 0.09	<0.001	<0.001	0.48 ± 0.05	0.52 ± 0.04	0.63 ± 0.07	<0.001	<0.001	0.10
Systolic blood pressure (mmHg)	122 ± 20	131 ± 21	129 ± 10	0.04	0.22	123 ± 22	117 ± 17	125 ± 6	0.29	0.91	0.34
Diastolic blood pressure (mmHg)	72 ± 9	76 ± 11	84 ± 6	0.006	0.002	75 ± 9	71 ± 6	77 ± 5	0.09	0.47	0.11
Total cholesterol (mg/L)	160 ± 43	210 ± 63	275 ± 45	0.002	<0.001	154 ± 61	182 ± 77	235 ± 55	0.02	0.009	0.67
Triglycerides (mg/L)	50 ± 17	57 ± 20	71 ± 47	0.42	0.17	54 ± 14	47 ± 15	71 ± 43	0.22	0.19	0.80
HDL cholesterol (mg/L)	48 ± 18	63 ± 21	70 ± 32	0.37	0.07	47 ± 20	39 ± 11	58 ± 23	0.10	0.22	0.37
LDL cholesterol (mg/L)	102 ± 40	136 ± 54	191 ± 80	0.02	0.006	96 ± 48	133 ± 81	163 ± 64	0.07	0.03	0.82
Fasting glucose (mmol/L)	4.56 ± 0.58	4.42 ± 0.55	4.93 ± 0.56	0.14	0.21	4.46 ± 0.50	4.85 ± 0.70	4.56 ± 043	0.20	0.61	0.04
2 h glucose (mmol/L)	5.46 ± 1.24	5.26 ± 1.00	8.17 ± 3.40	0.002	0.003	6.36 ± 1.21	6.67 ± 1.68	7.02 ± 1.14	0.48	0.25	0.05
Fasting insulin (mU/L)	4.66 ± 2.56	5.71 ± 4.39	8.95 ± 3.60	0.01	0.007	6.72 ± 2.69	9.56 ± 5.94	7.38 ± 3.26	0.44	0.59	0.06
**Euglycaemic clamp**											
M unadjusted (mg.min^−1^.kg^−1^)	10.0 ± 2.7	7.5 ± 2.4	5.2 ± 1.8	<0.001	<0.001	8.4 ± 2.2	7.6 ± 2.8	6.1 ± 1.7	0.02	0.01	0.13
M adjusted to lean mass (mg.min^−1^.kg^−1^)	11.6 ± 2.9	9.8 ± 3.3	7.8 ± 2.9	0.004	0.001	11.5 ± 2.7	11.1 ± 3.4	10.4 ± 3.1	0.70	0.35	0.27
**Fasting indices**											
Plasma insulin (mUI/mL)	4.7 ± 2.6	5.7 ± 4.4	8.9 ± 3.6	0.01	0.007	6.7 ± 2.7	9.6 ± 5.9	7.4 ± 3.3	0.44	0.59	0.06
Glucose/Insulin ratio (mmol/mUI)	25.5 ± 18.7	21.6 ± 13.3	11.6 ± 4.9	0.03	0.07	15.7 ± 12.0	13.6 ± 12.1	14.0 ± 6.8	0.50	0.69	0.30
HOMA-IR	0.95 ± 0.57	1.11 ± 0.89	2.00 ± 0.93	0.009	0.004	1.33 ± 0.56	2.08 ± 1.32	1.53 ± 0.65	0.34	0.47	0.02
FIRI	0.85 ± 0.51	1.01 ± 0.80	1.80 ± 0.84	0.009	0.004	1.20 ± 0.50	1.88 ± 1.19	1.37 ± 0.58	0.34	0.47	0.02
QUICKI	0.30 ± 0.15	0.19 ± 0.10	0.11 ± 0.03	0.01	0.10	0.15 ± 0.08	0.13 ± 0.08	0.13 ± 0.03	0.37	0.46	0.32

BMI: body mass index; HDL: high density lipoprotein cholesterol; LDL: low density lipoprotein cholesterol; HOMA-IR: Homeostasis Model Assessment for insulin resistance; FIRI: fasting insulin resistance index; QUICKI: quantitative insulin sensitivity check index.

### Insulin sensitivity across obesity categories

A total of 79 participants (47 men) had complete data on all variables of interest. Table 1 shows insulin sensitivity levels from various indices across BMI categories. The M-value was low in obese men and women, with significant differences across categories of BMI among men but not among women, with however no evidence of interaction (p = 0.27). Fasting plasma insulin, FIRI, HOMA-IR were highest in obese and lowest in lean men with significant differences across BMI categories, and in linear fashions (all p ≤ 0.01 for linear trends); whereas among women, these were highest in overweight and lowest among lean, with no significant difference or linear trends across BMI categories (all p ≥ 0.34); resulting in significant heterogeneity across sex and BMI categories (all p ≤ 0.06 for interaction, Table 1). The glucose/insulin ratio was highest among lean men, without any trend in women (p =0.69 for linearity). The QUICKI index was lowest in obese men and women, and highest among lean men and women, with however, significant difference across BMI categories only in men (p =0.01), but not in a differential way (p = 0.32 for sex* and BMI interaction).

### Correlation between fasting indices, clinical surrogates, and clamp measures

**Figure 1:**
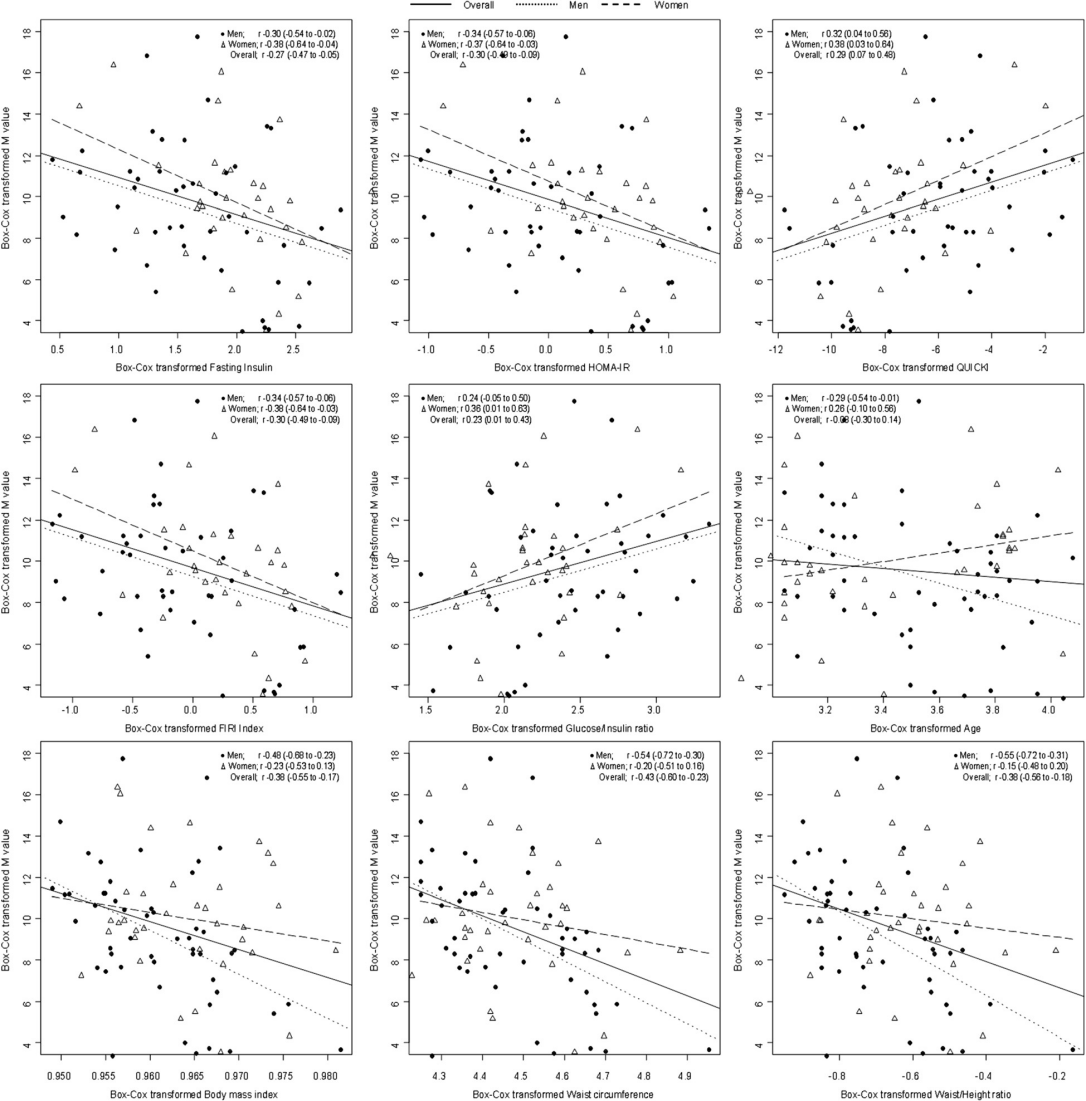
**Correlation between clamp-derived insulin sensitivity index (M value), and fasting indices and clinical surrogates of insulin sensitivity overall and in men and women.** Plots are based on the Box-Cox transformed values of insulin sensitivity of interest (x-axis) against the Box-Cox transformed M value (y-axis). Across figure panels, the filled circles are always for men and the point-up triangles for women. For each figure panel, the superimposed curves are the linear regression lines depicting the continuous association of indices of interest with M value overall (solid line) and for men (dotted lines) and women (broken lines). The accompanying correlation coefficient and 95% confidence intervals are also shown.

The correlation matrix of fasting indices and clinical surrogates of insulin sensitivity with clamp-derived index is described in Figure 1. In the overall sample, correlation coefficients (95% confidence interval) for fasting indices vs. clamp-derived index were −0.27 (−0.47 to −0.05) for fasting insulin, −0.30 (−0.49 to −0.09) for HOMA-IR, 0.29 (0.07 to 0.48) for QUICKI, −0.30 (−0.49 to −0.09) for FIRI and 0.23 (0.01 to 0.43) for glucose/insulin ratio. In analyses stratified by sex, the pattern was similar, with no difference by gender in the observed effects (all p > 0.60 for men vs. women comparisons). In the overall sample, correlations of fasting indices with clamp-derived index appeared to be significantly different for fasting insulin vs. QUICKI (p = 0.01) and glucose/insulin ratio (p = 0.03); HOMA-IR vs. QUICKI (p = 0.008) and glucose/insulin ratio (p = 0.02); QUICKI vs. FIRI (p = 0.008); and FIRI vs. glucose/insulin ratio (p = 0.02). The pattern was similar in men and women.

In the overall sample, correlation coefficients (95% confidence interval) clinical surrogates vs. clamp-derived index were −0.08 (−0.30 to 0.14) for age, −0.38 (−0.55 to −0.17) for BMI, −0.43 (−0.60 to −0.23) for waist circumference and −0.38 (−0.56 to −0.18) for WHtR. There was a trend toward stronger correlation in men than in women (Figure 1 and Table 2). The correlations of clamp with BMI, WC and WHtR were stronger than those with QUICKI, FIRI and glucose/insulin ratio (all p ≤ 0.0004), and not appreciably different to those with fasting insulin and HOMA-IR.

**Table 2:** Regression coefficients from multiple robust linear models for the prediction of clamp-derived index by fasting insulin sensitivity indices accounting for the potential effect of sex and adiposity

	**BMI**		**Waist**		**WHtR**		**BMI & WC**		**Fasting insulin**		**HOMA-IR**		**QUICKI**		**FIRI**		**Glucose/insulin**	
	**β**	**p**	**β**	**p**	**β**	**p**	**β**	**p**	**β**	**p**	**β**	**p**	**β**	**p**	**β**	**p**	**β**	**p**
Insuline sensitivity index	-	-	-	-	-	-	-	-	−1.472	0.01	−1.454	0.01	0.331	0.01	−1.454	0.01	1.761	0.03
Sex (female)	1.277	0.10	0.726	0.27	1.368	0.05	0.873	0.31	1.156	0.09	1.171	0.07	1.162	0.08	1.171	0.07	1.093	0.13
BMI	−200.3	0.0003	-	-	-	-	74.94	0.56	-	-	-	-	-	-	-	-	-	-
Waist	-	-	−8.964	<0.0001	-	-	6.130	0.30	−7.985	0.0002	−7.606	0.0004	−7.805	0.0002	−7.606	0.0004	−8.470	0.0002
WHtR					−8.1465	0.0002			-	-	-	-	-	-	-	-	-	-
R^2^	0.179		0.202		0.177		0.200		0.261		0.266		0.263		0.266		0.248	

BMI: body mass index; WHtR: waist-to-height ratio; β: standard regression coefficient; R^2^: coefficient of determination.

The best fitting multivariable model containing sex and each of the clinical surrogates was achieved with waist circumference (R^2^ = 0.202), in predicting clamp-derived index (Table 2). Adding any of the other clinical surrogates did not improve the fit of the model. The effect of each of the fasting indices on clamp-derived value was only marginal in multivariable models (Table 2). Altogether, each index, sex and waist circumference in the same model accounted for about 25 to 27% of the variability of clamp-derived measure of insulin sensitivity. Adding the interaction terms of indices with sex did not improve the fit of the models, while replacing WC with either BMI or WHtR resulted in less performing models. Across competing models (with each of the indices), WC was always the most significant and consistent determinant of clamp-derived index (Table 2).

### Classification agreement

The proportion of participants correctly ranked in cross-classification across thirds of clamp-derived and fasting insulin sensitivity indices and clinical surrogates is shown in Table 3. This proportion ranged from 27% to 51% overall, 25% to 68% in men, and 25% to 47% in women based on fasting indices. Significant kappa statistic were observed in the overall sample and in men for QUICKI vs. Clamp [kappa 0.24 (95% CI: 0.09-0.42) and 0.27 (0.05-0.46)], and for glucose/insulin ratio vs. clamp [0.26 (0.09-0.42) and 0.30 (0.08-0.51)]; but not in women, or for other fasting indices (Table 3). The best performing clinical surrogate was BMI [kappa 0.22 (0.05-0.38) in the overall sample and 0.36 (0.14-0.58) in men].

**Table 3:** Proportion of subjects correctly classified by each index using clamp-derived M thirds as reference insulin sensitivity categories

	**Men**					**Women**					**All subjects**				
**Insulin sensitivity category**	** Low**	** Medium**	** High**	** Total**	** Kappa (95% CI)**	** Low**	** Medium**	** High**	** Total**	** Kappa (95% CI)**	** Low**	** Medium**	** High**	** Total**	** Kappa (95% CI)**
**Using clamp as reference**	**N = 15**	**N = 16**	**N = 16**	**N = 47**		**N = 10**	**N = 11**	**N = 11**	**N = 32**		**N = 25**	**N = 27**	**N = 27**	**N = 79**	
Fasting plasma insulin	20%	44%	25%	14/47	−0.05 (−0.25 to 0.14)	30%	36%	9%	8/32	−0.13 (−0.33 to 0.10)	24%	41%	18%	22/79	−0.08 (−0.22 to 0.08)
Glucose/Insulin ratio	53%	56%	50%	25/47	0.30 (0.08 to 0.51)	50%	45%	45%	15/32	0.20 (−0.08 to 0.47)	52%	52%	48%	40/79	0.26 (0.09 to 0.42)
HOMA-IR	20%	37%	19%	12/47	−0.12 (−0.30 to 0.06)	20%	45%	18%	9/32	−0.08 (−0.31 to 0.15)	20%	41%	18%	21/79	−0.10 (−0.24 to 0.04)
FIRI	20%	37%	19%	12/47	−0.12 (−0.30 to 0.08)	20%	45%	18%	9/32	−0.08 (−0.30 to 0.16)	20%	41%	18%	21/79	−0.10 (−0.24 to 0.04)
QUICKI	53%	50%	50%	24/47	0.27 (0.05 to 0.46)	50%	36%	54%	15/32	0.20 (−0.04 to 0.47)	52%	44%	52%	39/79	0.24 (0.09 to 0.42)
Age	27%	9%	56%	15/47	−0.02 (−0.22 to 0.18)	10%	45%	27%	9/32	−0.08 (−0.32 to 0.16)	20%	26%	44%	24/79	−0.04 (−0.19 to 0.11)
BMI	53%	56%	62%	27/47	0.36 (0.14 to 0.58)	50%	27%	27%	11/32	0.01 (−0.23 to 0.25)	52%	44%	48%	38/79	0.22 (0.05 to 0.38)
Waist	53%	31%	56%	21/47	0.20 (−0.02 to 0.40)	50%	27%	18%	10/32	−0.03 (−0.26 to 0.22)	52%	30%	41%	32/79	0.11 (−0.06 to 0.27)
WHtR	53%	31%	62%	25/47	0.23 (0.01 to 0.43)	50%	36%	18%	11/32	0.01 (−0.22 to 0.25)	52%	33%	44%	34/79	0.14 (−0.02 to 0.31)

HOMA-IR: Homeostasis Model Assessment for insulin resistance; FIRI: fasting insulin resistance index; QUICKI: quantitative insulin sensitivity check index;

BMI: body mass index; WHtR: waist-to-height ratio.

## Discussion

This study aimed to validate a wide-range of fasting indices of insulin sensitivity against hyperinsulinemic euglycemic clamp among non-diabetic sub-Saharan Africans. We found that, although fasting indices of insulin sensitivity were strongly correlated with each other, they only displayed weak associations with clamp-derived measure of insulin sensitive, and consistently among men and women. These associations were further attenuated after adjustment for clinical markers of adiposity, which appeared to be significant determinants of insulin sensitivity in this population. However, accounting for the effect of adiposity (via waist circumference), sex and fasting indices explained just about 25% of the variability of insulin sensitivity in our sample. Among participants ranked by thirds of fasting indices, less than half fall within the corresponding category based on thirds of clamp-derived measure. Similar results were obtained when using clinical surrogates of insulin sensitivity. Our results are of importance given the rapid ongoing changes in physical activity and nutritional behaviors, resulting in increasing insulin resistance and consequential escalating rates of diabetes and obesity in SSA. Addressing these challenges would require larger-scale studies of T2DM or obesity, in which the use of examined indices may prove beneficial.

The relatively low levels of correlations between indices and clamp measures may be the result of a higher basal insulin secretion in people of African ancestry [17], which may have clouded the relationship between these indices and clamp-derived measure. Although a direct comparison of our findings with what would be obtained in a Caucasian population is not possible, our results after adjusting for sex and adiposity are similar to those reported by Pisprasert et al. [7] who found similar coefficients of correlation between clamp-derived insulin sensitivity index and HOMA-IR (0.266 vs. 0.290) as well as QUICKI (0.263 vs. 0.265). In overweight African-American premenopausal women, Alvarez et al. [6] reported similar coefficients of correlation between insulin sensitivity index and Glucose/Insulin ratio while the coefficient was slightly higher for fasting insulin (0.261 vs. 0.308) and HOMA-IR (0.266 vs. 0.309), and lower for QUICKI (0.263 vs. 0.249). In all groups assessed in our study, fasting insulin had comparable correlations to those observed with the more complex indices of insulin resistance (HOMA-IR, QUICKI or FIRI) with clamp insulin sensitivity. Thus, HOMA-IR, QUICKI, and FIRI may not necessarily provide better estimation of insulin sensitivity than that obtained from fasting insulin alone in individuals of SSA origin.

Very few studies have examined surrogate indices against clamp-derived measures in population from SSA. Existing studies have mainly been from South Africa and were generally restricted to a limited number of indices, contrary to our investigation that comprehensively examined a wide range of indices. Ntyintyane et al. [18], reported significant correlations between clamp-derived insulin sensitivity and log HOMA-IR (r = −0.34) and QUICKI (r = 0.41) among Black South Africans; which in major ways were similar to those from our study. However, their study was based on a smaller and more heterogeneous group of participants including people with coronary heart disease and or diabetes mellitus [18]. Furthermore, they did not adjust clamp-derived M-values for lean body mass, and used lower insulin infusion rates (40 mU/m^2^/min), which may have resulted in incomplete suppression of hepatic glucose production (HGP) in participants with high BMI.

Our study has some limitations. Firstly, the study circumstances may not fully mimic real-life situation in the sense that participants were under controlled conditions before testing. Secondly, although we included more participants than any previous study from SSA, our sample size may still be small to uncover some significant associations. However, performing clamp studies in larger sample is logistically challenging in any setting. Thirdly, although we used a high-rate insulin infusion, in the absence of concomitant use of radiolabeled glucose tracer during the clamp studies, we are unable to confirm that we achieved total suppression of hepatic glucose production. Fourthly, we used two different methods (absorptiometry and bioimpedance) to measure fat body mass. This approach could possible induce differential measurement error, with possible, by likely marginal effect on the relationship of fasting indices and clamp-derived measures [19]. Lastly, we excluded heavy smokers (>20 cigarettes per day) from our sample. We made this choice because of uncertainties surrounding the association between smoking and insulin resistance from studies in Caucasians [20-22]. There have been suggestions that smoking 24 cigarettes per day increases energy expenditure by about 10% [23], which in turn could be associated with a lower body mass index, a key determinant of insulin resistance.

The strengths of our study relate primarily to our reliance on robust methods including: 1) the use of the reference method for estimating insulin sensitivity (hyperinsulinemic euglycemic clamp); the use of a systematic sequential- as opposed to a random approach to OGTT and the clamp studies; 3) the use of multiple and robust statistical approaches to assess the validity of surrogate indices. Furthermore, our cohort included a wide range of insulin sensitivity/BMI, as BMI is known to influence the relationship between surrogate indices and direct measurements of insulin sensitivity [24]. In the absence of established cut-off to diagnosed insulin resistance in this population, we purposefully refrained from assessing the predictive utility of indices based on arbitrary cut-offs derived from our sample.

## Conclusions

In conclusion, fasting indices for insulin sensitivity are very modest determinants of clamp-derived measure of insulin sensitivity among non-diabetic sub-Saharan Africans. Furthermore, these indices appear not to perform better than common clinical measures of adiposity, nor to add significant predictive information to knowledge from non-invasive clinical measure in predicting insulin sensitivity. More research efforts are needed to identify in this setting to identify affordable fasting indicators which, singly or in combination may improve the accuracy of insulin sensitivity prediction. Predictive research in other settings has demonstrated the usefulness of routine clinical parameters in predicting the occurrence of diabetes mellitus or cardiovascular diseases, which are all long-term consequence of insulin resistance [25,26]. Adapting knowledge from those studies to the African setting will significantly improve the prevention and control of insulin resistance related status, without necessarily measuring insulin sensitivity, which at present appear to be unreliable using advocated fasting estimators.

## Competing interests

The authors declare that they have no competing interests.

## Authors’ contributions

ES, JFG and JCM: conceived the study and design study, data collection and analysis, and drafting of the manuscript. APK: analysis of data and drafting of the manuscript. JBE, SC, JST, EVB, MSP, VS, VE, EL, OTD, BAT: data interpretation, editing and reviewing the manuscript. ASM and PB: study design, data collection, editing and reviewing the manuscript. All authors read and approved the final manuscript.

## Pre-publication history

The pre-publication history for this paper can be accessed here:

http://www.biomedcentral.com/1472-6823/14/65/prepub

## References

[B1] Danaei G Finucane MM Lu Y Singh GM Cowan MJ Paciorek CJ Lin JK Farzadfar F Khang YH Stevens GA Rao M Ali MK Riley LM Robinson CA Ezzati M Global Burden of Metabolic Risk Factors of Chronic Diseases Collaborating Group (Blood Glucose) National, regional, and global trends in fasting plasma glucose and diabetes prevalence since 1980: systematic analysis of health examination surveys and epidemiological studies with 370 country-years and 2.7 million participants Lancet 2011 378 31 40 21705069 10.1016/S0140-6736(11)60679-X

[B2] Finucane MM Stevens GA Cowan MJ Danaei G Lin JK Paciorek CJ Singh GM Gutierrez HR Lu Y Bahalim AN Farzadfar F Riley LM Ezzati M Global Burden of Metabolic Risk Factors of Chronic Diseases Collaborating Group (Body Mass Index) National, regional, and global trends in body-mass index since 1980: systematic analysis of health examination surveys and epidemiological studies with 960 country-years and 9.1 million participants Lancet 2011 377 557 567 21295846 10.1016/S0140-6736(10)62037-5 PMC4472365

[B3] Whiting DR Guariguata L Weil C Shaw J IDF diabetes atlas: global estimates of the prevalence of diabetes for 2011 and 2030 Diabetes Res Clin Pract 2011 94 311 321 22079683 10.1016/j.diabres.2011.10.029

[B4] DeFronzo RA Tobin JD Andres R Glucose clamp technique: a method for quantifying insulin secretion and resistance Am J Physiol 1979 237 E214 E223 382871 10.1152/ajpendo.1979.237.3.E214

[B5] Muniyappa R Lee S Chen H Quon MJ Current approaches for assessing insulin sensitivity and resistance in vivo: advantages, limitations, and appropriate usage Am J Physiol Endocrinol Metab 2008 294 E15 E26 17957034 10.1152/ajpendo.00645.2007

[B6] Alvarez JA Bush NC Hunter GR Brock DW Gower BA Ethnicity and weight status affect the accuracy of proxy indices of insulin sensitivity Obesity (Silver Spring) 2008 16 2739 2744 18927554 10.1038/oby.2008.437 PMC2779542

[B7] Pisprasert V Ingram KH Lopez-Davila MF Munoz AJ Garvey WT Limitations in the use of indices using glucose and insulin levels to predict insulin sensitivity: impact of race and gender and superiority of the indices derived from oral glucose tolerance test in African Americans Diabetes Care 2013 36 845 853 23223406 10.2337/dc12-0840 PMC3609485

[B8] Kengne AP Limen SN Sobngwi E Djouogo CF Nouedoui C Metabolic syndrome in type 2 diabetes: comparative prevalence according to two sets of diagnostic criteria in sub-Saharan Africans Diabetol Metab Syndr 2012 4 22 22650602 10.1186/1758-5996-4-22 PMC3407752

[B9] Alberti KG Zimmet PZ Definition, diagnosis and classification of diabetes mellitus and its complications. Part 1: diagnosis and classification of diabetes mellitus provisional report of a WHO consultation Diabet Med 1998 15 539 553 9686693 10.1002/(SICI)1096-9136(199807)15:7<539::AID-DIA668>3.0.CO;2-S

[B10] Friedewald WT Levy RI Fredrickson DS Estimation of the concentration of low-density lipoprotein cholesterol in plasma, without use of the preparative ultracentrifuge Clin Chem 1972 18 499 502 4337382

[B11] Duncan MH Singh BM Wise PH Carter G Alaghband-Zadeh J A simple measure of insulin resistance Lancet 1995 346 120 121 7603193 10.1016/s0140-6736(95)92143-5

[B12] Matthews DR Hosker JP Rudenski AS Naylor BA Treacher DF Turner RC Homeostasis model assessment: insulin resistance and beta-cell function from fasting plasma glucose and insulin concentrations in man Diabetologia 1985 28 412 419 3899825 10.1007/BF00280883

[B13] Wallace TM Levy JC Matthews DR Use and abuse of HOMA modeling Diabetes Care 2004 27 1487 1495 15161807 10.2337/diacare.27.6.1487

[B14] Katz A Nambi SS Mather K Baron AD Follmann DA Sullivan G Quon MJ Quantitative insulin sensitivity check index: a simple, accurate method for assessing insulin sensitivity in humans J Clin Endocrinol Metab 2000 85 2402 2410 10902785 10.1210/jcem.85.7.6661

[B15] Box GEP Cox DR An analysis of transformations J Royal Stat Soc B (Methodological) 1964 26 211 252

[B16] Kent JT Tyler DE Vardy Y A curious likelihood identity for the multivariate t-distribution Commun Stat Simul Comput 1994 23 441 453

[B17] Haffner SM D’Agostino R Saad MF Rewers M Mykkanen L Selby J Howard G Savage PJ Hamman RF Wegenknecht LE Bergman RN Increased insulin resistance and insulin secretion in nondiabetic African-Americans and Hispanics compared with non-Hispanic whites. The Insulin Resistance Atherosclerosis Study Diabetes 1996 45 742 748 8635647 10.2337/diab.45.6.742

[B18] Ntyintyane L Panz V Raal F Gill G Comparison between surrogate indices of insulin sensitivity and resistance, and the hyperinsulinaemic euglycaemic glucose clamp in urban South African blacks with and without coronary artery disease Diab Vasc Dis Res 2010 7 151 157 20382779 10.1177/1479164109360271

[B19] Fogelholm M Van Marken LW Comparison of body composition methods: a literature analysis Eur J Clin Nutr 1997 51 495 503 11248873 10.1038/sj.ejcn.1600448

[B20] Henkin L Zaccaro D Haffner S Karter A Rewers M Sholinsky P Wagenknecht L Cigarette smoking, environmental tobacco smoke exposure and insulin sensitivity: the Insulin Resistance Atherosclerosis Study Ann Epidemiol 1999 9 290 296 10976855 10.1016/s1047-2797(99)00003-4

[B21] Chiolero A Faeh D Paccaud F Cornuz J Consequences of smoking for body weight, body fat distribution, and insulin resistance Am J Clin Nutr 2008 87 801 809 18400700 10.1093/ajcn/87.4.801

[B22] Eliasson B Attvall S Taskinen MR Smith U The insulin resistance syndrome in smokers is related to smoking habits Arterioscler Thromb 1994 14 1946 1950 7981184 10.1161/01.atv.14.12.1946

[B23] Hofstetter A Schutz Y Jéquier E Wahren J Increased 24-hour energy expenditure in cigarette smokers N Engl J Med 1986 314 79 82 3941694 10.1056/NEJM198601093140204

[B24] Kim SH Abbasi F Reaven GM Impact of degree of obesity on surrogate estimates of insulin resistance Diabetes Care 2004 27 1998 2002 15277430 10.2337/diacare.27.8.1998

[B25] Noble D Mathur R Dent T Meads C Greenhalgh T Risk models and scores for type 2 diabetes: systematic review BMJ 2011 343 d7163 22123912 10.1136/bmj.d7163 PMC3225074

[B26] van Dieren S Beulens JW Kengne AP Peelen LM Rutten GE Woodward M van der Schouw YT Moons KG Prediction models for the risk of cardiovascular disease in patients with type 2 diabetes: a systematic review Heart 2012 98 360 369 22184101 10.1136/heartjnl-2011-300734

